# Influence of internal migration on the use of reproductive and maternal health services in Nepal: An analysis of the Nepal Demographic and Health Survey 2016

**DOI:** 10.1371/journal.pone.0216587

**Published:** 2019-05-09

**Authors:** Naba Raj Thapa, Sunil Adhikari, Pawan Kumar Budhathoki

**Affiliations:** The 2018 DHS Fellow, Department of Population Studies, Ratna Rajya Laxmi Campus, Tribhuvan University, Nepal; Tribhuvan University Institute of Medicine, NEPAL

## Abstract

**Background:**

Internal migration has been an integral part of socioeconomic transformation in a country. Migrants are a vulnerable group for access to the reproductive and maternal health services. Very little is known regarding the role of internal migration on the use of reproductive and maternal health services in Nepal. This study examines the effect of internal migration on the use of reproductive and maternal health services in Nepal.

**Methods:**

The data for this study were extracted from the 2016 Nepal Demographic and Health Survey (2016 NDHS). The study population is women age 15–49. The sample population is different for modern contraceptive use than for Antenatal care (ANC) visits and place of delivery. The sample population for modern contraceptive use is restricted to the 8,811 (weighted) women who are currently married. The total analytic sampled population for ANC visits and place of delivery is 3,220 (weighted) women. The study used descriptive and logistic regression analysis, with three outcome measures: current use of modern contraception; at least four ANC visits; and place of delivery.

**Results:**

Sixty-eight percent women were internal migrants. Forty-four percent of eligible women reported current use of modern contraception, 71% of women made at least four ANC visits, about 9% of women made 8 or more ANC visits and 58% of women delivered in a health facility. Our findings show that modern contraceptive use is significantly higher among urban non-migrant women and urban-to-urban migrants. Urban-to-urban migrant women and rural-to-urban migrant women have significantly higher odds of attending at least four ANC visits for the most recent birth compared with rural-to-rural migrant women. Women who moved between urban areas, women who moved from an urban to a rural area, women who moved from a rural area to an urban area and urban non-migrants are significantly more likely to deliver in a health facility compared with women who moved between rural areas.

**Conclusion:**

The differentials of use of reproductive and maternal health services by migration status may need consideration during program planning to improve women's reproductive and maternal health services in Nepal.

## Introduction

Nepal has been experiencing a rapid increase in the volume of internal migration over the last 30 years. The volume of inter-district lifetime migrants in Nepal increased from 9% in 1981 to 15% in 2011. Similarly, the volume of inter-regional lifetime migrants increased from 7% in 1981 to 10% in 2011 [1, 2]. Census data show that most internal migration in Nepal occurs from Hill to Terai regions and from rural to urban areas [3].

Internal migration has been an integral part of socioeconomic transformation in a country [4]. Migration has positive as well as negative consequences. The 2011 census data shows that rural-to-rural and rural-to-urban migration are the prominent migration streams in Nepal. During the last decade, migrants who moved from rural to rural areas decreased from 68% in 2001 to 59% in 2011 and migrants who moved from rural to urban areas increased from 26% in 2001 to 36% in 2011 [2]. Rural-to-rural and rural-to-urban migration in Nepal involves a large segment of population. Migration from rural areas has been deeply connected with socioeconomic, political, cultural and environmental conditions. Regulating internal migration can bring benefits for countries as well as individuals. Poorly managed internal migration, however, can result in various difficulties, including reproductive and maternal health problems [5].

Types of migration and health are interconnected in different ways. Female migrants are a vulnerable group for reproductive and maternal health problems, which pose different challenges to meeting their medical needs [6]. The negative effects of migration are more prominent for women than men [7], although the effect of migration on migrant's health is complex, and variations exist between migrant groups. The effect of migration on health outcomes varies according to who migrates, when they migrate, where they migrate from, where they migrate to, and what health outcome is measured [8].

In the literature, three theoretical explanations have been used to explain the cause of differentials in use of reproductive and maternal health services between migrant and non-migrant women: selection, adaptation, and disruption. The selection theory explains that, outside of natural disasters, migrants are not randomly selected, but rather are selected on the basis of background characteristics such as age, education, and occupational status [9–11]. Adaptation is the process of change in the migrant’s attitudes, values, culture, beliefs, and behaviors in line with their new environment. Adaptation in the new environment depends on the migrant’s socio-cultural and economic background [10, 12, 13]. Migration disruption refers to separation of spousal partners, family members, and relatives immediately after migration [14, 15]. Migration selection may be either positive or negative. Positively selected migrants are more educated and economically well, whereas negatively selected migrants are less educated, poor economic status, and unskilled. Positively selected migrants have better access to reproductive and maternal health services. Migrants who moved to new area have more likely to face number of challenges in adjusting new social environment. Migration disruption may lead to delay in the use of reproductive and maternal health services, since migration selectivity, adaptation and disruption affect women's access to reproductive and maternal health services.

Data from past five Nepal Demographic and Health Survey (NDHS) show that the use of modern contraceptive methods has increased, from 26% in 1996 to 43% in 2016. Over the past 20 years, the percentage of women who had at least four ANC visits during their pregnacy has also increased, from 14% in 2001 to 69% in 2011. Similarly, the percentage of women who delivered in a health facility has increased significantly, from 8% in 1996 to 57% in 2016 [16].

A study in Peru shows that rural migrant women face different problems in their health needs compared with non-migrant women. Rural migrant women are less likely to use modern contraceptive methods and to receive appropriate ANC compared with urban non-migrant women. The study further pointed out that rural migrant women are more likely to have only a primary level of education, to have no health insurance, and to be in the lowest wealth category compared with urban migrants and urban non-migrants [17].

A study in Myanmar showed that female internal migrants had better reproductive health outcomes compared with non-migrants [18]. Women in Bangladesh who migrated to urban areas were significantly less likely than non-migrants to use reproductive health services related to pregnancy and ANC or to use modern contraceptives [19]. Studies in Chinese contexts found that internal migrants used ANC significantly less than non-migrants [20]. A hospital-based cross-sectional study on ANC use showed that rural-to-urban migrant women did not receive adequate antenatal care. Inadequate use of ANC is associated with low socioeconomic status and with demographic factors [21]. A study in Guatemala showed that current use of modern contraceptives was positively associated with women’s education. The study also found that urban non-migrants were more likely to use modern contraception compared with rural non-migrants [22].

Women’s use of reproductive and maternal health services is determined by various factors. The study hypothesized that migration status of women and socio-demographic factors would influence use of reproductive and maternal health services. Modern contraceptive use, ANC visits, and place of delivery are outcomes of reproductive and maternal health services, which are influenced by migration status of women. Women’s age, education, caste/ethnicity, occupation, and wealth status are socio-demographic factors that also effect the use of reproductive and maternal health services outcomes through migration status. Therefore, this study aimed to examine the effects of internal migration on the use of modern contraceptives, at least four ANC visits and health facility.

This study is important to investigate the effect of internal migration on the use of reproductive and maternal health services among the women age 15–49 years in Nepal. In addition, the findings of the study would be useful to policy makers in designing interventions to mitigate differentials in the use of reproductive and maternal health services due to internal migration of women.

## Research questions

1. Is there an association between internal migration and modern contraceptive use among currently married women age 15–49?,
2. Is there an association between internal migration and at least four ANC visits for most recent birth in the last five years?, and
3. Is there an association between internal migration and delivered in a health facility for the most recent birth in the last five years?.

## Data and methods

### Data and sampling design

Data for this study were extracted from the 2016 Nepal Demographic and Health Survey (NDHS). The 2016 NDHS is the fifth and most recent Demographic and Health Survey conducted in Nepal, and is a nationally representative population-based survey. The survey was conducted under the aegis of the Ministry of Health, Government of Nepal, with the financial support of the United States Agency for International Development (USAID) and technical assistance from ICF through The DHS Program. The survey involved the use of two-stage sampling in rural areas and three-stage sampling in urban areas. In rural areas, wards were selected as primary sampling units (PSUs) and households were selected from the sample PSUs. In urban areas, wards were selected as PSUs, one enumeration area (EA) was selected from each PSU, and households were selected from the sample EAs. In this survey, 11,473 households were selected for the sample, of which 11,040 households were interviewed. Likewise, 13,089 women age 15–49 were identified for individual interviews, and 12,862 were successfully interviewed, yielding a 96% response rate.

### Sample population

The study population is women age 15–49. In order to ensure uniform comparisons, we excluded women who were temporary visitors to the surveyed household (n = 410) and women who stated that they had moved to the area from abroad (n = 685). The total weighted number of internal migrants and non-migrant women eligible for the study is 7,876 and 3,791 respectively.

The sample population is different for modern contraceptive use than for ANC visits and place of delivery. The sample population for modern contraceptive use is restricted to the 8,811 (weighted) women who are currently married are using any modern contraceptive methods. Unmarried women; household visitors and those who moved to the district form abroad are excluded form analysis. The total weighted number of analytic sampled population for ANC visits and place of delivery is 3,220 women. The sample population for at least four ANC visits is restricted to women how attended at least four ANC visits for the most recent birth in the five years preceding the survey. Similarly, the sample population for delivered in health facility is comprised women who delivered in a health facility for the most recent birth in the five years preceding the survey. Women whose most recent birth was longer ago than the year they moved to the district (n = 283) are excluded from the analysis.

### Variable selection

#### Outcome variables

**Use of modern contraception:** This variable is coded as a binary outcome for whether using modern contraception, including male sterilization, female sterilization, injectable, intrauterine devices (IUD), pill, implants, male condoms, lactational amenorrhea, and emergency contraception. Traditional and folkloric method users were classified as non-users.

**ANC visits:** The World Health Organization (WHO) recommends that women attend at least four ANC visits as a necessary part of maternal health care [23]. The number of ANC visits is coded as 'yes' for women with at least four ANC visits before their most recent (4+ ANC visits) in the last five years and coded as 'no' if there were fewer than four visits.

**Place of delivery:** This variable is categorized into the binary outcome 'health facility' and 'home/elsewhere'. The place of delivery is coded as 'yes' for health facility and 'no' for home/elsewhere.

#### Independent variables

**Migration variables:** The 2016 NDHS asked the question: "How long have you been living continuously in the current place of residence?" Those women who answered "always" are treated as "non-migrants", while those women who reported "number of years lived in current place of residence" are considered as "migrants" if they changed place of residence across district boundaries. A further question was asked on previous place of residence before moved to current place of residence. This information was used to generate six categories of migration status: urban-to-urban (U-U), urban-to-rural (U-R), rural-to-urban (R-U), rural-to-rural (R-R), urban non-migrant, and rural non-migrant. A woman who reported previous place of residence as rural and current place of residence as urban is classified as a rural-to-urban migrant. Similarly, a woman who reported always lived in the current place is considered as non-migrant, either urban or rural.

**Covariates:** Covariates included in this study were women’s age, education, caste/ethnicity, occupation, and wealth quintile. Women’s age is categorized into four groups—15–19, 20–29, 30–39, and 40–49. In the NDHS, caste/ethnicity has 11 categories. However, for a better explanation, we grouped the variable into five categories where Brahman/Chhetri included Hill Brahman, Hill Chhetri and Terai Brahman/Chhetri, Dalit included Hill Dalit and Terai Dalit, Janajati included Newar, Hill Janajati and Terai Janajati, and Muslim included Muslim and others. The variable occupation has eight categories. This variable is grouped in three categories—not working, agriculture, and non-agriculture, where the non-agriculture group included professional/technical, clerical, sales/services, skilled manual, unskilled manual, and others. Wealth quintile is a composite measure of household living standard. Data on household assets were collected in the NDHS. Household wealth index was constructed using household assets data, including ownership of a number of consumer items ranging from a television to a bicycle or car, and such housing characteristics as sources of drinking water, sanitation facilities, fuel used for cooking, room used for sleeping, types of materials used for flooring, and ownership of agricultural land [16].

### Data analysis

This study employed three levels of statistical analysis. First, descriptive statistical techniques were used by their relative percentage at the univariate level with to analyze selected variables of study population of women age 15–49. Second, chi-square (χ^2^) analysis was performed to assess association between outcome variables and independent variables, and selected covariates at the bivariate level. Results are considered significant at p<0.05. Third, adjusted logistic regression analysis were employed to examine the net effects of migration status on the three outcome variables (modern contraceptive use; at least four ANC visits; and health facility delivery) with adjusting selected covariates—women’s age, education, caste/ethnicity, occupation, and wealth quintile. Multicollinearity was checked before logistic regression. In multiple logistic regression analysis, we adjusted women's age, education, caste/ethnicity, occupation and wealth quintile. The results are presented in adjusted odds ratios (AOR). All the analyses were performed using STATA version 15.1. The complex sample design of the NDHS was taken into consideration.

### Ethics statement

Data from the 2016 Nepal Demographic and Health Survey (2016 NDHS) was used for this study. We downloaded data from https://www.dhsprogram.com/data/available-datasets.cfm with register as DHS data users. The DHS program accessed us the data after reviewing our research proposal. We accepted terms and conditions attached with data sharing policy. As the data were obtained from the records, we could not consent women for accessing their records. We retrieved data of women age 15–49 for this study. Information collected from respondents under the DHS had been anonymized. The ethical approval for the survey was obtained by ICF International Institutional Review Board (IRB) and the Ethical Review Board of Nepal Health Research Council. All the respondents had provided verbal informed consent prior to each interview. Further approval for this study was not required since data are available in public domain.

## Results

### Socio-demographic and economic characteristics

Table 1 shows that 68% women are internal migrants. One-third of the women are rural-to-urban migrants and are age 20–29 and about one-quarter are age 30–39, while 21% are age 15–19, and 20% are age 40 and above. Regarding education, 33% of women have no education, 26% have completed secondary level of education, and 25% have attained at least a School leaving certificate (SLC) level of education. Janajati (37%) and Brahman/Chhetri (33%) are the dominant caste/ethnicity of the study population. Nearly two-thirds of women (63%) are urban residents. In considering occupation status, 32% of women are not working and about 48% are working in agriculture. The study population is more or less evenly distributed across the wealth quintiles, with 18% of women in the lowest quintile and 22% in the highest. In all, a majority of the study population is under age 30, educated, of Janajati or Brahman/Chhetri caste/ethnicity, urban, and engaged in agriculture.

**Table 1:** Percentage distribution of women aged 15–49 by migration status and background variables, Nepal DHS 2016.

Variables	Percent	Total (n)
**Migration status**		
Migrants	67.5	7876
Non-migrants	32.5	3791
**Migration streams**		
Urban-to-Urban	10.0	1166
Urban-to-Rural	2.2	259
Rural-to-Urban	33.5	3912
Rural-to-Rural	21.8	2539
Urban non-migrants	19.7	2304
Rural non-migrants	12.7	1487
**Age group**		
15–19	20.7	2413
20–29	33.4	3901
30–39	26.2	3062
40–49	19.6	2290
**Education**		
No education	32.8	3826
Primary	16.6	1938
Secondary	25.9	3026
School leaving certificate (SLC) and above	24.7	2877
**Ethnicity**		
Brahman/Chhetri	33.0	3845
Terai caste	13.3	1552
Dalit	12.4	1444
Janajati	37.0	4317
Muslim	4.4	509
**Occupation**		
Not working	31.5	3677
Non-agriculture	20.3	2374
Agriculture	48.1	5615
**Wealth quintile**		
Lowest	17.7	2063
Second	19.8	2312
Middle	19.9	2321
Fourth	21.0	2451
Highest	21.6	2519
**Total**	**100.0**	**11667**

### Descriptive analysis

Table 2 presents the associations of modern contraceptive use, ANC visits, and place of delivery with migration status and other selected background characteristics. Five of the six variables—women’s age, education, caste/ethnicity and occupation—are significantly associated with modern contraceptive use. Migration status and wealth quintile do not have statistically significant associations with modern contraceptive use. Urban non-migrant women have the highest level of contraceptive use, followed by urban-to-urban migrant women. The use of modern contraception ranges from 41% among urban-to-rural migrants to 48% among urban non-migrants.

**Table 2:** Percentage of eligible women age 15–49 using modern contraception, who had at least four ANC visits, and delivered their most recent birth in the five years preceding the survey in a health facility, according to migration status and selected background characteristics, Nepal DHS 2016.

Variables	Modern contraceptive use (n = 8811)		4+ ANC visits (n = 3220)		Delivered at health facility (n = 3220)	
	% (95% CI)	χ^2^ p-value	% (95% CI)	χ^2^ p-value	% (95% CI)	χ^2^ p-value
**Migration status**						
Urban-to-urban	45.6 (41.7–49.6)	0.059	89.9 (83.4–94.1)	< 0.001	87.5 (80.3–92.4)	< 0.001
Urban-to-rural	40.7 (33.8–48.0)		71.9 (58.5–82.2)		66.5 (52.6–78.0)	
Rural-to-urban	44.7 (42.1–47.3)		76.4 (72.8–79.6)		67.8 (63.1–72.1)	
Rural-to-rural	40.9 (37.8–44.1)		62.4 (57.7–66.9)		42.7 (38.1–47.4)	
Urban non-migrants	48.4 (43.9–52.9)		76.0 (70.4–80.9)		64.0 (55.5–71.6)	
Rural non-migrants	43.7 (39.4–48.1)		61.0 (52.5–68.8)		38.6 (30.4–47.4)	
**Age**						
15–19	15.7 (12.4–19.7)	< 0.001	77.8 (71.4–83.1)	< 0.001	69.7 (62.2–76.3)	< 0.001
20–29	32.6 (30.3–34.9)		72.8 (70.0–75.4)		59.2 (55.6–62.8)	
30–39	52.6 (49.9–55.4)		67.1 (62.5–71.4)		52.1 (47.0–57.0)	
40–49	57.3 (54.3–60.1)		45.6 (35.5–56.0)		31.3 (21.6–43.0)	
**Education**						
No education	53.4 (50.8–56.0)	< 0.001	52.6 (48.0–57.2)	< 0.001	35.1 (31.0–39.5)	< 0.001
Primary	43.4 (40.3–46.5)		64.8 (60.2–69.2)		47.9 (42.9–52.9)	
Secondary	34.8 (32.3–37.3)		79.6 (76.4–82.5)		68.9 (64.6–72.9)	
SLC and above	35.3 (32.4–38.2)		92.8 (90.4–94.7)		84.6 (80.9–87.7)	
**Caste/ethnicity**						
Brahman/Chhetri	41.3 (39.1–43.5)	< 0.001	81.0 (77.1–84.3)	< 0.001	69.6 (64.5–74.2)	< 0.001
Terai caste	45.1 (41.6–48.8)		61.4 (54.3–68.1)		43.9 (37.4–50.7)	
Dalit	44.2 (40.4–48.1)		63.1 (57.1–68.7)		46.7 (40.6–52.9)	
Janajati	47.6 (44.7–50.5)		72.6 (68.1–76.6)		60.4 (54.9–65.6)	
Muslim	29.5 (22.8–37.2)		56.5 (41.9–70.1)		42.2 (30.3–55.1)	
**Occupation**						
Not working	36.5 (34.0–39.0)	< 0.001	69.9 (65.4–74.0)	0.001	61.4 (57.0–65.5)	< 0.001
Non-agriculture	46.3 (43.7–48.9)		81.3 (75.8–85.7)		75.2 (68.3–81.1)	
Agriculture	47.5 (45.0–50.0)		69.0 (65.8–72.0)		49.7 (45.9–53.5)	
**Wealth quintile**						
First	42.6 (39.2–45.9)	0.538	56.9 (51.8–61.8)	< 0.001	35.0 (29.6–40.8)	< 0.001
Second	45.8 (42.8–48.9)		67.7 (63.1–72.1)		46.8 (42.0–51.5)	
Middle	44.8 (41.7–48.0)		70.3 (64.7–75.3)		58.6 (53.6–63.5)	
Fourth	43.0 (40.0–46.2)		77.9 (73.5–81.8)		70.8 (65.9–75.3)	
Highest	43.6 (40.7–46.6)		90.4 (85.9–93.6)		89.9 (85.0–93.4)	
**Total**	**44.0 (42.4–45.6)**	** **	**71.1 (68.5–73.6)**	** **	**57.6 (54.4–60.7)**	** **

Women age 40–49 have the highest percentage of modern contraceptive use (57%), and women age 15–19 have lowest percentage (16%). Regarding education, 53% of women with no education are currently using modern contraception, followed by women with a primary education (43%). The use of modern contraceptives has an inverse relationship with women’s level of education. This could be due to the high level of sterilization among less educated women. Looking at caste/ethnicity, Janajati women have the highest proportion of modern contraceptive use (48%). In contrast, Muslim women (30%) and Brahman/Chhetri women (41%) have relatively low levels of modern contraceptive use. There are significant associations between women’s occupation and modern contraceptive use. Levels of modern contraceptive use are higher among women engaged in an agriculture occupation (48%) than women engaged in non-agriculture (46%) and non-working women (37%). There are no significant differences in use of modern contraception by wealth quintile.

Eight variables showed a highly significant (p<0.001) association with at least four ANC visits in the chi-square analysis. Table 2 shows that 71% of women made at least four ANC visits for the most recent birth. A high proportion of urban-to-urban migrant women (90%) made at least four ANC visits, while the lowest proportion was among rural non-migrant women (61%).

The study found that younger women are more likely to make at least four ANC visits compared with older women. About three-quarters (78%) of women age 15–19 made at least four ANC visits for their most recent birth compared with less than half (46%) of women age 40–49. There are significant associations between women’s education and ANC visits. As education level increases, the percentage of women making at least four ANC visits increases. About half (53%) of women with no education made at least four ANC visits compared with 93% of women who attained SLC and above education. Regarding caste/ethnicity, Hill Brahman/Chhetri women have the highest proportion making at least four ANC visits, followed by Janajati, Dalit, and Terai caste women. Muslim women have the lowest proportion making at least four ANC visits (57%). A large proportion of women engaged in a non-agricultural occupation (81%) reported at least four ANC visits. The proportion of women with at least four ANC visits ranges from 57% in the lowest wealth quintile to 90% in the highest wealth quintile.

Overall, 58% of migrant women delivered in a health facility. Use of a health facility for the most recent birth is higher among women of urban-to-urban migrants (88%) and lower among women of rural non-migrants (39%). The study shows that younger women are more likely to deliver in a health facility than older women. Seventy percent of women age 15–19 delivered in health facility compared with 31% of women age 40–49. There is a positive association between women’s education and health facility delivery. Women with primary education have the lowest percentage of health facility delivery (48%), whereas women with SLC and over education have the highest percentage (85%). Considering caste/ethnicity, Brahman/Chhetri women have the highest percentage of delivery in a health facility (70%), followed by Janajati (60%). Muslim women have the lowest percentage of health facility delivery (42%). Seventy percent of urban women delivered in a health facility compared with 43% or rural women. Women engaged in a non-agricultural occupation have the highest percentage of delivery in a health facility. By wealth quintile, health facility delivery ranges from 35% in the lowest wealth quintile to 90% in the highest quintile.

### Multivariate analysis

#### The effect of migration status on modern contraceptive use

Table 3 shows that migration status (urban-to-urban and urban non-migrants) have significant associations with modern contraceptive use after controlling for other factors. The odds of modern contraceptive use are higher among urban-to-urban migrants (OR = 1.37, p<0.05) and urban non-migrants (OR = 1.29, p<0.05) compared with rural-to-rural migrants. Urban-to-rural migrants, rural-to-urban migrants, and rural non-migrants appear more likely to use modern contraception compared with rural-to-rural migrants. However, odds of modern contraceptive use are not statistically significant for these migration status.

**Table 3:** Adjusted odds ratio showing association between migration status and modern contraceptive use, ANC visits, and delivery in a health facility among women age 15–49, Nepal DHS 2016.

Variables	Modern contraceptive use (n = 8811)	4+ ANC visits (n = 3220)	Delivered in health facility (n = 3220)
	OR (95% CI)	OR (95% CI)	OR (95% CI)
**Migration status**			
Rural-to-rural	1.00	1.00	1.00
Urban-to-urban	1.37* (1.06–1.77)	1.89* (1.02–3.50)	2.74** (1.46–5.11)
Urban-to-rural	1.11 (0.81–1.53)	1.04 (0.58–1.88)	1.96* (1.12–3.42)
Rural-to-urban	1.20 (1.00–1.45)	1.35* (1.02–1.78)	1.92*** (1.43–2.58)
Urban non-migrants	1.29* (1.02–1.64)	1.40 (0.97–2.01)	1.60* (1.09–2.33)
Rural non-migrants	1.12 (0.94–1.34)	1.03 (0.74–1.42)	0.94 (0.66–1.33)
**Age**			
15–19	1.00	1.00	1.00
20–29	2.33*** (1.75–3.11)	0.67* (0.47–0.97)	0.47*** (0.32–0.69)
30–39	4.77*** (3.40–6.69)	0.64* (0.41–0.99)	0.42*** (0.27–0.66)
40–49	5.24*** (3.77–7.28)	0.43** (0.24–0.75)	0.35** (0.17–0.70)
**Education**			
No education	1.00	1.00	1.00
Primary	0.78** (0.67–0.91)	1.47** (1.14–1.89)	1.40* (1.07–1.82)
Secondary	0.63*** (0.53–0.74)	2.36*** (1.81–3.08)	2.29*** (1.75–3.01)
SLC and above	0.62*** (0.51–0.76)	5.74*** (3.72–8.85)	3.38*** (2.37–4.82)
**Caste/ethnicity**			
Dalit	1.00	1.00	1.00
Brahman/Chhetri	0.88 (0.72–1.07)	1.54* (1.09–2.17)	1.78** (1.22–2.61)
Terai caste	1.09 (0.86–1.38)	0.72 (0.47–1.11)	0.50*** (0.34–0.75)
Janajati	1.09 (0.91–1.32)	1.08 (0.78–1.49)	1.21 (0.86–1.70)
Muslim	0.48*** (0.32–0.71)	0.68 (0.37–1.26)	0.52* (0.30–0.92)
**Occupation**			
Not working	1.00	1.00	1.00
Non-agriculture	1.30** (1.11–1.53)	1.06 (0.76–1.49)	1.04 (0.75–1.45)
Agriculture	1.32** (1.12–1.56)	1.36* (1.06–1.76)	0.97 (0.77–1.21)
**Wealth quintile**			
First	1.00	1.00	1.00
Second	1.20* (1.00–1.44)	1.47** (1.11–1.94)	1.41* (1.04–1.91)
Middle	1.24* (1.01–1.52)	2.19*** (1.57–3.07)	3.35*** (2.32–4.82)
Fourth	1.20 (0.97–1.48)	2.81*** (1.94–4.06)	4.73*** (3.22–6.95)
Highest	1.19 (0.92–1.53)	3.64*** (2.21–6.00)	8.69*** (4.86–15.52)

*** p<0.001

** p<0.01

* p<0.05

As expected, married women age 40–49 have substantially higher odds of using modern contraception compared with women age 15–19. Women who attained SLC and above level of education have significantly lower odds of modern contraceptive use (OR = 0.63, p<0.001) compared with uneducated women. The odds of modern contraceptive use are 37% lower for women who attained a secondary level of education and 22% lower for women with a primary level of education compared with women who have no education. Regarding caste/ethnicity, the odds of modern contraceptive use are 52% lower for Muslim women compared with Dalit women (OR = 0.48, p<0.001). The odds of using modern contraceptives are 12% higher for Dalit women compared with Brahman/Chhetri women. Women engaged in agriculture or non-agricultural occupations have significantly higher odds of modern contraceptive use compared with non-working women. Women in the second and middle wealth quintiles have significantly higher odds of using modern contraception (OR = 1.20, p<0.05 and OR = 1.24, p<0.05 respectively) compared with women in the first wealth quintile.

#### The effect of migration status on ANC visits

Concerning the effect of migration status on ANC visits, Table 3 shows that urban-to-urban migrant women have statistically significant higher odds of ANC visits (OR = 1.89, p<0.05), as do rural-to-urban migrant women (OR = 1.35, p<0.05) compared with rural-to-rural migrant women. Considering other background variables, there are statistically significant association between women’s age, education, and wealth quintile and attending at least four ANC visits. Women age 40–49, 30–39, and 20–29 have statistically significant lower odds of ANC visits (OR = 0.43, p<0.01; OR = 0.64, p<0.05; and OR = 0.67, p<0.05 respectively) compared with young women age 15–19. The odds of receiving ANC are significantly higher among women with SLC and above education (OR = 5.74, p<0.001), secondary education (OR = 2.36, p<0.001), and primary education (OR = 1.47, p<0.01) compared with women with no education. Brahman/Chhetri women have 1.54 times higher odds of attending at least four ANC visits compared with Dalit women. Women engaged in agriculture have significantly higher odds of ANC visits compared with non-working women (OR = 1.36, p<0.05). There is also a statistically strong association between wealth quintile and ANC visits. Women in the highest wealth quintile are 3.6 times more likely to make at least four ANC visits compared with women in the lowest wealth quintile.

#### The effect of migration status on place of delivery

Table 3 shows that migration status have a statistically significant association with place of delivery after adjusting other factors. Urban-to-urban migrants, rural-to-urban migrants, urban-to-rural migrants, and urban non-migrants have statistically significant higher odds of facility delivery (OR = 2.74, p<0.001; OR = 1.92, p<0.001; OR = 1.96, p<0.05 and OR = 1.60, p<0.05 respectively) compared with rural-to-rural migrant women. Rural non-migrants have lower odds of health facility delivery (OR = 0.94) compared with rural-to-rural migrant women. Women age 30–39 and 40–49 have significantly lower odds (OR = 0.47, p<0.001 and OR = 0.42, p<0.001 respectively) compared with women age 15–19. More educated women are more likely to deliver in a health facility compared with less educated women. The odds of health facility delivery are significantly higher among women who attained an SLC and above level of education compared with women who have no education (OR = 3.38, p<0.001).

Brahman/Chhetri women are significantly more likely (OR = 1.78, p<0.01) to deliver in a health facility than Dalit women. The odds of health facility delivery are significantly lower among Terai caste and Muslim women (OR = 0.50, p<0.001 and OR = 0.52, p<0.05) compared with Dalit women. There is a significant association between wealth quintile and health facility delivery. Women in the highest wealth quintile have 8.69 times higher odds of delivering in a health facility compared with women in the first wealth quintile. A statistically significant association does not exist between women’s occupational status and health facility delivery.

## Discussion

The overall objective of the study is to investigate the effects of internal migration on the use of reproductive and maternal health outcomes in Nepal. The analysis has been drawn from the datasets of 2016 Nepal Demographic and Health Survey.

The Nepal Health Sector Strategy 2016–2021 aims to expand equitable access to high-quality family planning services, increase the availability of modern family planning methods, and satisfy the demand for family planning [24]. The level of contraceptive use has increased substantially over the past decade, from 29% in 1996 [25] to 53% in 2016 [16]. Our study shows that the use of modern contraception varies with migration status of women. The use of modern contraceptive methods is highest among urban non-migrants, followed by urban-to-urban migrant women. This variation in results could be due to selectivity of migration. Bivariate analysis indicates that migration status are not significantly associated with use of modern contraception. A study in Kenya reveals that rural-to-urban migrants and urban non-migrants are more likely to use modern contraceptive than rural non-migrants [11]. When all other factors are taken into consideration, urban non-migrants and urban-to-urban migrants are more likely to use modern contraception compared with rural-to-rural migrants. The odds of using modern contraception are higher among urban-to-urban migrant women, rural-to-urban migrant women, and urban non-migrant women compared with rural non-migrant women. The multivariate analysis indicated that women’s age, education, and occupation have a strong association with modern contraceptive use. This finding is consistent with previous studies [26].

By realizing the necessity of improved health care services during pregnancy, childbirth and after delivery for wellbeing of mother and child, the Government of Nepal has implemented various strategies, programs to improve maternal, and child health. Sixty-nine percent of women had at least four ANC visits in Nepal [18]. This study shows that urban-to-urban migrant women and rural-to-urban migrant women are more likely to make four ANC visits than rural-to-rural migrant women. Previous studies from Peru showed that rural migrant women were less likely to make ANC visits than urban non-migrant women [17]. Another previous study showed that migrants women were less likely to use antenatal services when compared to non-migrants [20]. The analysis further points out that other controlling variable have significant effect on at least four ANC visits. Women's age, education, and wealth quintile are strongly associated with ANC visits.

Place of delivery determines the welfare of mother and babies. Increasing the percentage of births delivered in health facilities reduces maternal death due to complications of pregnancy and childbirth. Fifty-seven percent of women deliver in a health facility at the national level in Nepal [16]. The study reveals that migrant women are more likely to deliver in a health facility compared with non-migrant women. This finding is consistent with a study in Myanmar [18].

Facility delivery is highly associated with urban-to-urban migrant women. Rural-to-rural migrant women have significantly lower levels of delivery in a health facility compared with urban-to-urban migrants, rural-to-urban migrants, and urban non-migrant women. The study shows that rural non-migrant women have less likely to use health facility delivery. Rural non-migrant and rural-to-rural migrant women are less educated than urban non-migrant and urban migrant women. Furthermore, life style in rural areas has characterized traditional and have cultural resistance to deliver at health facility. Results of adjusted logistic regression suggest that internal migration flow is an important factor that strongly affects delivery in a health facility. The results further show that some control variables—women’s age, education, caste/ethnicity, and wealth quintile are significant predictors of health facility delivery.

The results of adjusted odds ratio analysis indicate that women's age, education, caste/ethnicity, and wealth quintile play a strong role in the odds of delivering in a health facility. This finding is consistent with previous studies in Kenya [27], Nigeria [28], Ghana [29], and Nepal [30]. However, in our study occupation is not found to be statistically significant in their association with health facility delivery.

The effects of internal migration on the use of reproductive and maternal health services have been studied by comparison of six categories of internal migration flows. This study confirms that urban-to-urban migrant women and urban non-migrant women are more likely to use modern contraceptives compared with rural-to-rural migrant women. Urban-to-urban migrant women and rural-to-urban migrant women are more likely to receive four ANC visits compared with rural-to-rural migrants. Similarly, urban-to-urban, urban-to-rural, rural-to-urban, and urban non-migrant women are more likely to deliver in a health facility compared with rural-to-rural migrant women.

## Conclusions

Migration status is significantly but differentially associated with use of modern contraceptives, at least four ANC visits, and health facility delivery. Modern contraceptive use is popular among women who migrate between urban areas and women who are urban non-migrants. Women who migrate to an urban area are more likely to make at least four ANC visits. Women are more likely to deliver in a health facility if they migrated to an urban area or if they are urban non-migrants. This study confirms that women who migrate to an urban area and urban non-migrants take better advantage of use of reproductive and maternal health services than rural non-migrants and women who migrate to a rural area.

Women who are urban-to-urban migrants or rural-to-urban migrants, women who are educated, Brahman/Chhetri women, and women in the highest wealth quintile are better advantage group for attending at least four ANC visits. Women who are urban migrants, urban-to-rural migrants and urban non-migrants, have attained higher education, are Brahman/Chhetri, and are in the highest wealth quintile have more access to deliver in a health facility.

This study has various implications for policymakers, program implementers, and stakeholders that could help to improve women's reproductive and maternal health services in Nepal. The differentials of use of reproductive and maternal health services by migration status should be considered when designing programs to improve women's access to reproductive and maternal health services.
